# Data are inadequate to test whale falls as chemosynthetic stepping-stones using network analysis: faunal overlaps do support a stepping-stone role

**DOI:** 10.1098/rspb.2017.1281

**Published:** 2017-09-27

**Authors:** Craig R. Smith, Diva J. Amon, Nicholas D. Higgs, Adrian G. Glover, Emily L. Young

**Affiliations:** 1 Department of Oceanography, University of Hawai'i at Manoa, 1000 Pope Road, Honolulu, HI 96822, USA; 2 Life Sciences Department, Natural History Museum, Cromwell Road, London SW7 5BD, UK; 3 Marine Institute, University of Plymouth, Drake Circus, Plymouth PL4 8AA, UK

Kiel [1] provides an interesting analysis of connectivity among bivalve and gastropod assemblages at hydrothermal vents, cold seeps and whale falls, suggesting a role for sedimented vents as evolutionary stepping stones between vents and seeps, but providing no support for whale falls playing a similar role. We caution that the dataset for whale falls used in Kiel [1], as well as further available data, are insufficient for network analysis to yield conclusions regarding lack of connectivity between whale falls, vents and seeps. Although Kiel [1] listed some limitations of the study, we present several more below to highlight the weaknesses of the whale-fall analysis.

A global connectivity analysis based on presence–absence data [1] relies on the validity of at least two assumptions:

(i) each vent, seep or whale-fall site included in the analysis has been well sampled; i.e. if a taxon does not occur in the data from a particular site, it is because it is absent from that site, and not a consequence of undersampling. Thus, absence from the data can be assumed to provide ‘evidence of absence’; and
(ii) the sites representing a particular habitat type are broadly (in fact, globally) distributed, with a substantial sample size within regions (e.g. ocean basins) to avoid geographical biases. In other words, there should be multiple (well sampled) sites for each habitat type in all regions across the globe so that patterns within regions, and within and between habitats, can be revealed.

The datasets for vent and seep habitats in Kiel [1] may well meet these assumptions, with 32 and 37 apparently well-sampled sites, respectively, distributed broadly across the ocean basins. However, the deep-sea whale-fall dataset fails to meet these assumptions owing to a small number (7) of whale-fall sites, with only two sampled thoroughly for epifauna and sediment infauna. The sampling of sediment infauna is particularly important because sedimented vent habitats are identified as a linkage between vents and seeps. To make clear the limitations of the Kiel [1] dataset, it is important to discuss the whale-fall sites used in more detail.

Kiel [1] included data from seven purported deep-sea whale-fall sites. One of the ‘whale-fall’ sites is actually an artificially implanted cow carcass [2]. Cow carcasses are dramatically smaller, and have different bone sizes and characteristics (e.g. lipid content), than the carcasses of great whales (see Smith *et al.* [3] for a discussion of the important characteristics of great whale falls) so there is little reason to expect that the full suite of species responding to a large whale carcass would be found at a single cow carcass.

Two of the ‘whale-fall sites’ used in Kiel [1] off New Zealand and Iceland were actually isolated bones recovered in trawls from unobserved locations at the seafloor [4–6]. Because these trawl samples (i) collected only portions of whale skeletons, (ii) lack any indication that the bones came from a large intact whale fall, (iii) probably sustained significant loss of whale-bone fauna during the trauma of trawl recovery (approx. 90% of bone-associated species can fall off whale bones even when carefully collected by submersible, C. Smith 1988–2005, personal observations), and (iv) did not include sediment infauna, only ‘presence data’ have any meaning for these ‘whale-fall’ sites. In other words, the absence of a taxon from a site could easily be owing to insufficient sampling. The use of trawled bones as adequate samples of entire whale-fall sites (which can contain tens of thousands of individuals and hundreds of species distributed both in sediments and over hundreds of bones [3,7,8]), is similar to dredging rocks from a hydrothermal vent and interpreting the attached fauna as representative of the full suite of species likely to be found at the vent site.

Finally, the datasets for the whale-fall sites in Monterey Canyon [9] and the Southern Ocean [10] in Kiel [1] included little or no infaunal sampling. Since vesicomyid bivalves (a key vent–seep–whale-fall molluscan taxon) at whale falls typically live buried within sediments underlying the bones and can only be fully identified with substantial sediment-sampling effort [11,12], these two sites are also very likely undersampled, especially with respect to vesicomyids. In fact, very recent infaunal data from the Monterey canyon whale fall reveal the presence of vesicomyid genera also found at sedimented vents and seeps [13].

This leaves two well-sampled whale-fall sites on the northeast Pacific margin in Kiel's [1] ‘global’ analysis of connectivity among vent, seep and whale-fall habitats. The small number and restricted distribution of these whale-fall sites provides minimal opportunity to explore global connectivity patterns, yielding little basis for Kiel's [1, p. 1] statement that: ‘The hypothesis that decaying whale carcasses are dispersal stepping stones linking these environments is not supported.’

Although several other whale-fall sites, not included in Kiel [1], have been sampled well [12,13], the whale-fall dataset is still too sparse to support a global network analysis [3]. However, shared habitats (e.g. sulfidic hard substrates, sediments and bacterial mats [3]), taxa and phylogenetic histories do implicate whale falls as ecological and evolutionary stepping stones for deep-sea reducing habitats such as vents and seeps. For example, one northeast Pacific whale fall harbours 10 genera also known from seeps (Annelida, Dorvilleidae: *Paurougia*, *Ophryotrocha*, *Schistomeringos*, *Exallopus*; Mollusca, Mytilidae: *Idas*; Vesicomyidae: *Archivesica*, *Pliocardia*, *Calyptogena*; Hyalogyrinidae: *Hyalogyrina*; Arthropoda, Isopoda: *Illyarachna*) and eight genera also known from vents (Annelida, Dorvilleidae: *Parougia*, *Ophryotrocha*, *Exallopus*; Polynoidae: *Bathykurila*; Mollusca, Mytilidae: *Idas*; Vesicomyidae: *Archivesica*, *Calyptogena*; Hyalogyrinidae: *Hyalogyrina*) [12]. Smith & Baco [7] report 10 species found on northeast Pacific whale falls that also occur at hydrothermal vents, and 20 species that also occur at seeps. On a whale skeleton in the abyssal South Atlantic, Sumida *et al.* [14] collected four genera of annelids shared with hydrothermal vents and/or cold seeps. Many of the genera and species shared between whale falls, vents and seeps are annelid worms which constitute a substantial portion of the diversity in deep-sea chemosynthetic habitats (e.g. [15–17]), suggesting that a full network analysis of faunal connectivity across deep-sea chemosynthetic habitats should include the Annelida.

The faunal overlaps across whale falls, vents and seeps, and the role of whale falls as ecological stepping stones, may well have been greater before the vast reduction of whale populations and the loss of whale falls resulting from human whaling activities [18]. Palaeo-ecological and phylogenetic studies of taxa associated with deep-sea vents and seeps also provide evidence for evolutionary connectivity with whale falls (reviewed in Smith *et al.* [3]), including the occurrence of basal clades of bathymodiolin mussels at whale falls [19] and indications of adaptive radiation at whale falls of taxa common at vents and seeps (e.g. the annelids in Siboglinidae [20] and Dorvilleidae [21,22]).

Because vents, seeps and whale-fall fauna share taxa and phylogenetic histories, there is a strong need for intensive sampling of deep-sea whale-fall communities (including the sediment infauna) in multiple ocean basins to support network analyses of the type conducted by Kiel [1]. This will allow us to fully elucidate global connectivity patterns among these deep-sea reducing habitats, and evaluate the roles that whale falls have played in supporting biodiversity and maintaining other ecosystem functions across the global ocean [23].
